# Progress Veterinary Science

**Published:** 1886-07

**Authors:** 


### Progress of Veterinary Science.

Research into the Union. of Tendons Mr. R. W. Parker and Prof. Horsley, have been investigating the question whether perfect reunion of tendons can occur when the ends are separated as far as possible. This question is one of very considerable practical surgical interest, in connexion with the orthopædic replacement of distorted parts, since it has been till the present time a matter of dispute whether the method of putting up a limb operated upon by tenotomy in a position of complete extension is or is not justifiable. As such a method presents many advantages over the older and more gradual treatment it seemed to Mr. Parker that the question ought to be decided by definite experiment.

Prof. Horsley divided the Achillis tendon in four dogs by the usual subcutaneous proceeding, and then fixed the limb in complete flexion by a suitable immobile splint of collodion and gauze. The animals being etherized, suffered no pain from the trifling operation, etc. A careful examination, both macroscopic and microscopic, by Mr. Parker, has in every case revealed the important fact that complete reunion of the tendon subsequently occurred, so that the point in question may now be considered as definitely settled in favor of the rapid method of treatment referred to above. The details of the above experimental results will be published shortly by Mr. Parker.

Coagulation of the Blood.—Dr. Wooldridge has been engaged on the study of this subject.

The results obtained have been communicated to the Royal Society, and are partly of such a nature as not to permit of an abstract being given.

Perhaps the most interesting result obtained has been the Isolation of a Substance from perfectly fresh Animal tissues, the injection of a solution of which into the blood of an animal causes instant death.

The most convenient sources of this substance are the Testes and Thymus gland of the Calf; but it also occurs in other tissues.

It causes death by producing intravascular fibrinous coagulation. Thus, into the jugular vein of a rabbit, a solution containing about ½ gram of the substance was injected. The animal died within a few seconds, and the whole vascular system was completely thrombosed, both sides of the heart being completely filled with clot, as was the case with the Aorta, Vena cava, Vena porta, all the mesenteric Veins, Carotids, Iliacs. etc.

This result is not due to the admixture of any fibrin ferment with the substance. The latter is a proteid body in combination with Lecithin : it entirely loses its power by being subjected to action of artificial gastric digestion for two or three hours.

The action of the substance is perfectly constant, the extent of the intravascular clotting depending on the amount of substance injected, so that a certain dose is necessary in order to kill.

It is singular that in dogs a moderate dose causes death by Thrombosis of the portal vein, this being a perfectly constant lesion, the sudden plugging of this vein giving rise to a fatal fall in blood-pressure.

In addition to causing intravascular coagulation the substance prevents he coagulation of shed blood,—that is to say, blood drawn off, after injecion of a moderate quantity, remains fluid for a very long period, ultimately, however, clotting.

There is reason to hope that this substance may be made practically useful, and full details will appear shortly.

Research into the Function of the Thyroid Gland.—In December, 1884, Prof. Victor Horsley showed that the thyroid gland was intimately connected with the process of mucin metabolism, that if the thyroid gland in monkeys was removed with antiseptic precautions (the same ensuring healing of the wound in three days) the consequences to the animal were—(1) symptoms of general nervous disturbance evidenced by tremors, paroxysmal convulsions, functional paralysis, mental hebetude, and finally complete imbecility ; (2) profound anæmia coupled with leucocytosis; (3) all the symptoms of the disease discovered within the last decade and termed myxœdema ; (4) that just as in the acute form of the disease just named there was found to be a great accumulation of mucin in the connective tissues throughout the body (mucinoid degeneration) and in the blood, and as a consequence the same post mortem appearances ; (5) that at the same time there was a great activity in the mucin-secreting glands, and, further, that the parotid gland under these abnormal circumstances secreted mucin in large quantity, the gland-cells at the same time disintegrating.

During the past year I have confirmed my previous observations and greatly extended them, and have firm basis for my original opinion that the function of the thyroid gland is indispensable to the higher animals, and that it is duplex in function, since in the first place, it regulates the formation of mucin in the body ; and, in the second place, it aids in the manufacture of blood-corpuscles. My researches during the past year (1885) have been directed towards the investigation of (1) the circumstances which influence the course of the extensive disturbance of general nutrition which follows the loss of the gland ; (2) the direct effect of the said fall in nutrition upon the nerve-centres ; and (3) the hæmapoietic function of the gland.

(1) I find that the determining factor par excellence of the value of the gland as regards its influence on the general metabolic processes of the animal is age. The effect of removing the gland in the young animal is the rapid appearance of violent nerve-symptoms and death in a few days ; in a rather older animal, *i. e.*, a one-year old dog, the symptoms are less violent, later in their appearance, and the animal survives perhaps for a fortnight or three weeks ; in a very old animal the removal of the gland simply hastens the torpor of old age ; these observations refer to dogs and cats. In the higher animals, monkeys, the operation on a young individual produces the same result as in a young dog, but, as I showed last year, an older animal, if kept under ordinary circumstances, will survive for six or seven weeks, dying at the end of that time of myxœdema. On the whole, therefore, it appears that the thyroid gland is of extreme importance when tissue metabolism is most active, and that it diminishes in value as the senile state advances. Huschke has shown that the relative weight of the thyroid body to the body-weight is greatest at birth, that it rapidly diminishes during the next few weeks, and that it steadily decreases as age advances. Finally, the structural degeneration of the gland in old age is well known. It is clear, therefore, that the gland plays an important and constant part in the metabolism of the body : I desire here to draw special attention to the fact that the symptoms of old age, namely, wasting of the actively functional parenchymatous tissues, atrophy, and falling out of the hair, decay of the teeth, dryness and harshness of the skin, tremors, etc., are exactly the most prominent features of the myxœdematous state, whether it occurs naturally in the human being, prematurely, as in cretinism, or artificially, as in my experiments on monkeys. It is, perhaps, well to remark here that. as might have been foreseen. the previous state of nutrition of the body determines to a large extent the rapidity of onset and the course of the symptoms.

The next circumstance of extreme importance which influences the course of the symptoms is the Temperature at which the animals are kept after the gland has been removed. I showed last year that one of the most striking features of the fall of nutrition which follows the loss of the gland was a steady diminution of the body heat. this suggested to me a line of research which has yielded a striking result. I have kept another series of animals (on whom I have performed thyroidectomy under the conditions above stated) at a constant temperature of 90° F.,* and when they exhibited any nerve-symptoms, *i. e.*, tremors, etc., placed them in a hot-air bath at a temperature of 105° F. The effect of this has been to lengthen the duration of life (in all but very young animals) four or five times the extent of that observed in the first series. Instead of living four to seven weeks they now live as many months. At the same time several additional facts of importance are noted, and the symptoms before referred to are so modified as to require the addition of a third stage of the two described in 1884. (These

*In my first experiment, (1884), the animals were kept at a temperature varying from 60° to 70°, F.

observations refer solely to monkeys). The animals kept under the extra high temperature above noted thus pass through three stages—1. neurotic; 2, mucinoid; 3. atrophic. I have said that the neurotic stage under these circumstances may be scarcely marked, or if the nerve-symptoms occur, and the animal be put in the hot-air bath, they soon disappear. Next, the animal lives through the mucinoid stage, *i.e.*, myxœdematous condition and arrives in the third stage, the atrophic. Now, the symptoms of the second stage are just as much subdued as those of the first; there is no excessive secretion of mucus, the parotid glands do not swell, and the post-mortem examination does not reveal the extensive mucinoid degeneration observed in the first series. Finally, the third, atrophic, stage into which the animal passes is evidenced by great emaciation, functional paresis and paralysis, imbecility, falling blood-pressure and temperature, with death by coma.

I am disposed to regard this fact of the animals passing through these neurotic, mucinoid stages, and dying at the end of the atrophic, as the key to the observation that cretins in whom the thyroid gland is very slowly destroyed, and very chronic cases of myxœdema do not exhibit much mucinoid degeneration.

(2), I will now briefly enumerate the direct effect of the fall of nutrition produced by the loss of the thyroid gland on the nerve-centres: *a*. Effect on cortex. The tetanus * obtained by stimulating the cortex is remarkably changed (even as soon as one day after the thyroidectomy in a dog, which exhibited violent symptoms in twenty-four hours) by the fact of the fall (when the current was shut off) being as sudden as that observed on stimulating the corona radiata. Next, that the tetanus in a more advanced case is soon exhausted, the curve approaching the abscissa soon after the initial rise; at the same time the curve is followed by clonic epileptoid spasms, which, however, are soon exhausted. Stimulation of the corona radiata and spinal cord also gave the customary tetanus, which, like that of the cortex, was rapidly exhausted. These stimulations of the nerve-centres suppressed the thyroid tremors just as voluntary movements do. Another evidence of the changes in the cortex is the frequency with which continuous stimulation will evoke the appearance of clonic spasms on the original tetanic curve, the latter not being followed by epilepsy when the current is shut off.

(*b*). Effect on the spinal cord. The tetanus obtained by stimulation of the spinal cord, like that of the cortex, rises slowly to the highest point, and then steadily falls towards the abscissa although the stimulation is maintained; and when the current is shut off the muscle completely relaxes, having absolutely lost its tone, and this tonic paralysis is not recovered for from 10 to 15 seconds. Stimulation of the spinal cord to fatigue, after some time has elapsed so as to produce exhaustion of the preliminary tetanus, evokes a tremor of eight to ten per second.

Tracings from an old animal (cat) which had survived the operation some months, and also from a dog and monkeys, in which case the symptoms had been very severe for some days, exhibited only a very feeble tetanus in the

---

† Graphically recorded according to method described by Prof. Schafer and myself. ("Proc. Roy, Soc.," Dec. 10, 1885).

former instance, and practically no reaction at all in the latter ; this being the ultimate state of depression of function which the nerve-centres had arrived at.

(3) I have thought it as well to add to the anatomical and physiological proofs I gave last year of the thyroid gland being a hæmapoietic structure by counting the number of corpuscles in the blood of the thyroid artery and vein respectively. After discounting any possible alteration in the relative number of the corpuscles in the two vessels by changes in the fluid constituent of the blood which may have happened in the gland, the much greater number of corpuscles in the vein (x 7 per cent.) confirms the deductions drawn from my previous observations.

To sum up : the function of the thyroid gland appears to me to be two-fold, as already suggested, viz.: (1) Control of mucin metabolism ; (2) Hæmapoiesis. The metabolic processes in the body may be regarded broadly as resulting in Construction and Destruction. The products of destruction are the waste-products of tissue change, and being, as such, harmful to the organism, are cast out by the excretory organ. It appears to be that the thyroid gland aids in excretion of mucinoid substances or their precursors, not of course by excretion properly speaking, that is, casting them out from the body, but by metamorphising them into some other form which is useful to the system. That this process, whatever it is, is of vital importance to the young mammal (seeing that interference with it causes death in a few days) is obvious, and such as it is, the loss of it is distinctly connected with the appearance of the disease known as myxœdema, cretinism and senile degeneration. Finally, this defect in the circle of metabolism determines the appearance of so-called functional disorders of the nervous system.

RESEARCH INTO THE PATHOLOGY OF THE DISEASE IN DOGS, KNOWN AS CANINE CHOREA, A SEQUELA OF DISTEMPER. By Prof. V. Horsley.—Up to the present time much information has been collected concerning the Pathological Anatomy of this condition, but owing to each observer having but two or three animals to examine, such information has been of but little value, owing to wide discrepancies between the results as published by the different investigators. Besides the obvious importance of deciding, if possible, this debateable point, it seemed to me extremely important to investigate closely the Pathology of the Nerve-Symptoms of this disorder by the most recent methods. From a number of experiments (seven) I have confirmed the observations of previous workers to the effect that the disorder of mechanism is situated in the spinal cord, and have further established the accuracy of this opinion by showing that the artificial restoration of the tonus of the muscles does not reproduce the rhythmical twitching. I have also shown that the influence of the higher centres upon the lower is, to a considerable extent, elucidated by the stimulation (for method see below) of various portions of the motor tract in this disease from the cortex downwards. From the experiments it would appear that the influence of the cortex upon the spinal cord is to increase (double) the rate of rhythmical discharge in the latter without, however, increasing the force of the latter at all, consequently, that the rate being doubled, the energy of each discharge is halved. In addition to this fact I have noted others of a similar nature (published in detail in the "Brown Lectures," 1885), which illustrate the interaction of the disorganized nerve-centres. The second half of the investigation (namely, the anatomical portion) has resulted in establishing harmony between the discrepant observations before referred to, by showing that the different appearances seen by various observers were really successive stages of the malady. From examination of very severe cases I have been able to go further than this, and I have demonstrated that the final stage of the "inflammatory" condition of the spinal cord is sclerosis, and sclerosis identical in every respect with disseminated sclerosis in man. I may be, perhaps, excused if I here point out that the significance of this fact lies in the demonstration that sclerosis may be the final stage of an "inflammatory" condition.

The methods referred to above were the same as those described by Prof. Schafer and myself in the "Proc. Roy. Soc.", December, 1885.

Disease of the Circulaory Organs in Animals.—Mr. Bland Sutton read a paper before the Pathological Society of London, giving a brief account of the various diseases which affect the organs of circulation in animals. Lesions of the heart and vessels were by no means of so frequent occurrence as in man—indeed, they were comparatively rare.

Inflammation of the pericardium arose from much the same causes, and presented similar characters to the same disease in man. It might arise from injuries, extension from pleurisy, simple or tubercular (perlsucht), parasites and rheumatic fever (?). It was well known to veterinarians that horses suffered from rheumatic fever and that pericarditis was a frequent complication. Mr. Sutton was strongly of the opinion that mammals which frequent the water, such as the hippopotamus, beaver, otter and others, suffered from this disease. It was in these animals that most of the uncomplicated cases of pericarditis occurred; and many days before death they lie about, abstain from entering the water, and when stirred, manifest signs of pain. Of the different varieties of pericarditis, specimens were shown from a lizard and a peacock (parasitic); from a beaver, an antelope and a monkey (rheumatic); from a tiger, coatimundi, etc., arising from extension, and one specimen from a monkey where the pericardium was enveloped by a mass of lympho-sarcoma, giving rise to effusion into the pericardial cavity. It was interesting to note, according to Rayer, that Galen described the first case of pericardial effusion, and that it occurred in a monkey. The most interesting cases of traumatic pericarditis were chiefly found in cows. When cropping the grass these animals frequently took up foreign bodies, such as pieces of wire, needles, etc. These sharp bodies were checked by the psalterium, and diverted from their course; they might pierce the coats of the stomach, pass through the diaphragm, and enter the pericardium, where they gave rise to violent inflammation. This condition was well known to veterinary surgeons.

The milk-white patch was very frequent in some birds and monkeys as a consequence of pressure from rickets, but the deformity induced by the latter disease led to a very serious condition of the heart, which might be best termed "flexion." If a young monkey who had died from rickets be frozen and vertically bisected, it would be found that the spinal column was in a condition of curvature (cat's back); this was often so severe that the sternum also became flexed, and a "knuckling" took place and squeezed the heart between the spine and sternum, and flexed it. This led to atrophy of the wall of the right ventricle, and in some cases this was so marked that the outer boundary of the cavity only consisted of the visceral layer of the pericardium. The condition would probably be found in children if carefully looked for in extreme cases of rickets.

Affections of the valves, endocarditis, vegetations and calcifications occurred occasionally, leading to incompetency and its consequences. Specimens were exhibited from sheep, Coypu rats, deer, antelopes, buffalo and a bear.

Diseases of the vessels were exceedingly rare, atheroma was found fairly often in the posterior aorta of the horse, and verminous aneuryism occurred in probably 90 per cent. of adult asses; excluding these examples, diseases of the vessels were exceedingly rare. Among the very great number of wild animals examined, twice only was atheroma detected—in a buffalo and in a zebu, the sacred ox of India. As a consequence of this, true aneuryism was very rare, and Mr. Sutton only knew of about three genuine cases in wild animals.

Verminous aneurism, although so common in asses and fairly frequent in horses, was yet confined to the domesticated animals; he had not found it in any of the wild members of the group. Fortunately the disease did not affect man, and this explained the little interest it excited in the medical profession, although the presence of these worms in the tunic of an artery was known to Ruysch as far back as 1665.

The last affection to notice was arterio capillary fibrosis, which affected the medium-sized arteries of the horse and cattle, and, as in man, was associated with chronic interstitial nephritis. Perhaps the most interesting fact in this connection was that which indicated the rarity of atheroma in animals. It had been noted previously by writers, but few had adduced any number large enough to make a generalizatian. The infrequency of the disease may be due to the abscence of alcoholism, syphilis and arterial strain.—*Veterinarian.*

NEPHRITIS IN LAMBS.—At a meeting of the Pathological Society in London Mr. Roger Williams exhibited specimens of acute nephritis in Lambs. The disease began soon after birth with difficulty in walking, the new-born falling down and lying on one side, sometimes with choroid movements, without loss of consciousness, without difficulty in breathing or cough; sucking was not impaired, nor the appetite. The disease appeared to attack the lambs of ewes imported from Scotland, and especially those whose female parents were served by a ram who was far from vigorous, having to spend his strength on too many ewes. At all events, it was found that if a ram only served ten ewes the progeny were vigorous, and did not become affected with the disease. Mr. Williams had examined two lambs, one that died of the disease and another killed at three weeks old. The urine was albuminous and acid, whereas it should have been alkaline. There were no renal casts, crystals, pus or blood. The capsules of the kidneys stripped off easily, exposing a surface studded with congested stellate veins. The cortex was swollen and soft, pale and yellowish in color, whilst the pyramids were firm and deep red in color. The disease was an acute tubal nephritis; the tubular epithelium was much degenerated, not staining with log-wood, whilst the interstitial tissue was normal. The changes in both cases were identical, though one was in a more advanced condition than the other. Nothing abnormal was detected in any of the organs, and the spinal cord was healthy. Some of the lambs were born with the disease and soon succumbed. Dr. Pye-Smith said the normal reaction of lambs' urine whilst suckling was acid.—*Veterinary Journal.*

